# Study of Thermal Behavior of Moxa Floss Using Thermogravimetric and Pyrolysis-GC/MS Analyses

**DOI:** 10.1155/2021/6298565

**Published:** 2021-02-16

**Authors:** Min Yee Lim, Xinyue Zhang, Jian Huang, Liang Liu, Yutang Liu, Baixiao Zhao, Hui Hu, Furong He, Junjie Xie, Dongsheng Qiu

**Affiliations:** ^1^International School, Beijing University of Chinese Medicine, Beijing 100029, China; ^2^School of Acupuncture-Moxibustion and Tuina, Beijing University of Chinese Medicine, Beijing 100029, China; ^3^Dongfang Hospital, Beijing University of Chinese Medicine, Second-affiliated Hospital, Beijing 100078, China; ^4^China Academy of Launch Vehicle Technology, Beijing 100076, China; ^5^Tobacco Research Institute of Chinese Academy of Agricultural Sciences, Qingdao 266101, China; ^6^School of Chinese Medicine, Beijing University of Chinese Medicine, Beijing 100029, China; ^7^School of Acupuncture-Moxibustion and Tuina, Fujian University of Traditional Chinese Medicine, Fujian 350122, China; ^8^Acupuncture Department, Xiamen Hospital, Affiliated Hospital of Beijing University of Chinese Medicine, Xiamen, China

## Abstract

Moxa floss is a type of biomass used as the main combustion material in moxibustion, a therapy that applies heat from moxa floss combustion to points or body areas for treatment. Safety concerns regarding moxa smoke have been raised in recent years. Since moxa floss is the source material in moxibustion, its thermal behavior and pyrolysis products would be related to the products formed in moxa smoke. This work aims to understand the thermal behavior of moxa floss and investigate the pyrolysis products generated from moxa floss combustion. Six commercial moxa floss samples of 3 storage years and 10 storage years, and of low, medium, and high ratios, were selected. The kinetic data from moxa floss combustion was carried out by a thermogravimetric analyzer. Pyrolysis-gas chromatography and mass spectroscopy using a gas chromatograph and mass spectrometer equipped with a pyroprobe were used to examine the pyrolysis products. Thermogravimetric profiles for all the samples were overall similar and showed a monotonic weight decrease. The range of intensive reaction temperature occurred between 150°C and 450°C, which was characterized by a major weight loss and accompanied by an exothermal degradation of the main components. The average ignition temperature for the samples of 3 and 10 storage years was 218.3°C and 222.6°C, respectively, which was lower than most herbaceous plants. The identified pyrolysis products include monocyclic aromatic hydrocarbons, polycyclic aromatic hydrocarbons, ketones, acids, and alkanes. All were of relatively low intensities of below 5% in relative abundance. No volatiles were detected in the samples of 10 storage years. The relatively low values of ignition temperature suggested that moxa floss is more combustible and can be ignited more easily than other herbaceous plants. This may explain why moxa floss has remained as the preferred material used for moxibustion over the years.

## 1. Introduction

Moxibustion is a traditional Chinese medicine therapy that burns moxa floss and uses the heat generated to apply to certain points or body areas for disease treatment. Many studies have reported the beneficial effects of moxibustion for health protection, prevention, and treatment of diseases [1]. With increasing global healthcare burdens such as escalating healthcare costs and persistent rise in chronic noncommunicable diseases, the usage of moxibustion has become more prevalent in recent years [2]. However, the safety of moxibustion has come under increased scrutiny, especially with reports related to adverse reactions associated with moxibustion [3], as well as reports of chemical components and pollutants in moxa smoke that are detrimental to human health, including inhalable particulate matter, formaldehyde, naphthalene, benzene, and methylbenzene [4, 5].

Moxa floss is a type of plant material produced after several rounds of pulverization and sifting from dried mugwort leaves of the species *Artemisia argyi* Levl. et Vant., *Artemisia princeps* Willd., and *Artemisia vulgaris* L [6, 7]. During moxibustion, it is reasonable to postulate that the chemical constituents of moxa floss are subjected to a wide range of temperature in the presence of varying concentrations of oxygen. The combustion of moxa floss can be considered a type of biomass pyrolysis. Its chemical compounds either distil out of moxa floss and transfer intact to moxa smoke, or undergo pyrolytic reactions where the products get transferred to smoke or undergo reactions with other smoke constituents. Consequently, it is important to understand the thermal behavior and pyrolysis of products that are generated during the combustion process.

Among various thermoanalytical techniques, thermogravimetric analysis (TGA) is a highly precise method useful for understanding the processes and mechanisms that take place during thermal degradation. TGA is a well-established method that allows the thermal and pyrolysis behavior of a product to be obtained in a straightforward manner [8] and has been used for nearly 50 years in the analysis of plant materials [9]. Pyrolysis is the process of decomposition of biomass with heat in inert atmosphere or vacuum nature at temperatures from 350 to 800°C. As the initial stage of the combustion and gasification process, pyrolysis plays a key role in determining ignition, flame stability, and products distribution [10]. During biomass pyrolysis, many reactions such as dehydration, depolymerisation, isomerization, aromatisation, decarboxylation, and charring occur in parallel or series [11]. It has been reported that each biomass sample shows unique characteristics under pyrolysis conditions because of difference in the ratios of their chemical constituents [12]. The pyrolysis process is thought to occur in 3 stages: in the first stage, moisture is removed from the biomass, after which the primary decomposition takes place, and lastly, secondary reactions (cracking and repolymerisation) occur [13]. The pyrolyzer coupled with gas chromatography/mass spectrometry (Py-GC/MS) is a commonly used technique in pyrolysis studies, in which the pyrolyzer provides different reaction conditions including different heating rates and final temperatures, and the GC/MS separate and identify the pyrolysis products [14].

Presently, most research is focused on moxa smoke and its chemical compounds [4, 5, 15–17]. Not much research has been done to understand the thermal behavior of moxa floss during combustion. Given that moxa floss is the source material used in moxibustion, its thermal behavior and pyrolysis products would invariably be related to the products formed in moxa smoke and have a direct bearing on the safety of this therapy. In this study, the thermal behavior and pyrolysis products of commercially available moxa floss were investigated. This work aims to understand the thermal behavior of moxa floss and to investigate the pyrolysis products generated from moxa floss combustion.

## 2. Materials and Methods

### 2.1. Materials

Six commercial moxa floss samples of 3 storage years and 10 storage years, and of low, medium, and high ratios, were selected in this study. The general information of the samples is shown in Table 1. Ratio refers to the weight of the starting material (dried mugwort leaves) to the weight of the finished product (moxa floss) in kilogram after processing. A moxa floss product of high ratio would be finer as compared to that of low or medium ratio. The samples were kept in 20°C and 50% relative humidity for 24 h before the experiment was conducted. All the samples were dried at 100°C in advance.

### 2.2. Thermogravimetric Analysis

The kinetic data from moxa floss combustion was carried out by a thermogravimetric analyzer (SDT Q600, TA, USA) using an inert atmosphere of high purity nitrogen gas with a flow rate of 50 mL/min. About 6 mg of each sample was placed in a platinum crucible without lid. The temperature ranged from room temperature to 1100°C. The heating rate used was 10°C/min.

### 2.3. Pyrolysis-Gas Chromatography and Mass Spectroscopy (Py-GC/MS)

Pyrolysis-gas chromatography and mass spectroscopy (Py-GC/MS) was performed using a PerkinElmer Clarus 680-SQ 8T gas chromatograph and mass spectrometer equipped with a Pyroprobe 6150. The procedure and conditions of experiment used in this study were as follows: Py-GC/MS analyses were conducted on whole samples. About 0.5 mg of sample was flash-pyrolyzed with an initial temperature of 50°C for 5 min, then at a heating rate of 5°C/min to 300°C. The sample was then loaded into a thin quartz tube and inserted into a filament coil in the pyroprobe. The pyrolysis unit was attached to the gas chromatograph and the pyrolysis products were transferred directly to a capillary column (RXItm-5 ms Restek 30 m, 0.25-mm i.d.; 0.25-mm film thickness). Helium was used as a carrier gas. The GC oven was cryogenically cooled to 40°C with liquid nitrogen. This temperature was held for 1 min, then increased at a rate of 4°C/min to 300°C, and held for 10 min. The mass spectrometer was operated in the electron-impact mode at 70 eV. The data of Py-GC/MS were processed using Turbomass6.0 software.

## 3. Results and Discussion

### 3.1. Thermogravimetric Analysis

**Figure 1:**
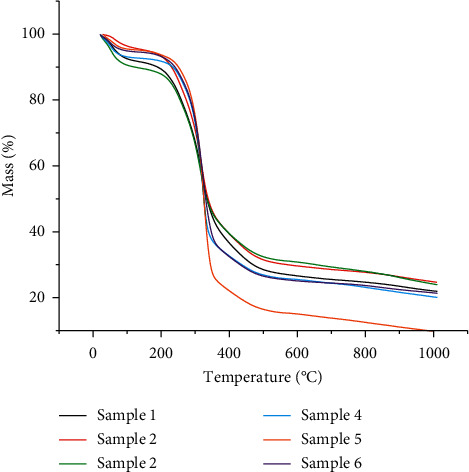
TG curves of the six moxa floss samples.

**Figure 2:**
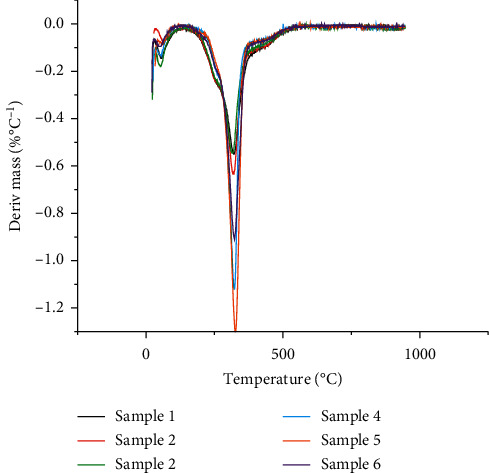
DTG curves of the six moxa floss samples.

The pyrolysis behaviors of moxa floss at the heating rate of 10°C/min were obtained. The TGA profiles of the samples and their derivative thermogravimetric profiles (DTG) are shown in Figures 1 and 2, respectively.  Table 2 lists the combustion characteristic parameters of the samples, including mass loss at temperature below 120°C, ignition temperature, peak temperature, burnout temperature, and residual weight at 900°C.

For all the samples, the TGA profiles were overall similar. They showed a monotonic weight decrease and could be divided into 3 stages. The first stage occurred below 120°C and was characterized by the loss of moisture and release of light volatiles in the samples. Studies have shown the volatile oil in *Artemisia argyi* folium to consist of monoterpenes, sesquiterpenes, alcohols, and aromatic compounds, varying in concentrations from 0.02% to 1.23% of the total content, depending on the geographical location and harvesting time [18, 19]. The second stage reflected the main thermal degradation in the combustion process, which took place between 150°C and 450°C and was characterized by a major weight loss and an exothermal course of degradation of the main components. The average ignition temperature for the samples of 3 and 10 storage years was 218.3°C and 222.6°C, respectively. The peak temperature, which is defined as the temperature at which the maximum rate of mass loss occurs [20], was at an average temperature of 318.8°C and 323.5°C for the samples of 3 and 10 storage years, respectively. A recent study found the ignition and peak temperature of moxa floss from Qichun, Hubei province, to be 266.1°C and 318.5°C, respectively [21]. The peak temperature was consistent with that of the 3 storage years samples in this study, but the ignition temperature of Qichun moxa floss was higher. The ignition temperature of a substance is defined as the least temperature at which the substance starts to combust and is greatly dependent on the chemical composition of the biomass [22]. The difference in ignition temperature might be due to a difference in the production area of the moxa floss, resulting in disparity in the content and composition of the chemical components, thus affecting the activation energy needed for combustion. Nonetheless, the average ignition temperature of 218.3°C and 222.6°C found in this study was still lower than other herbaceous plants reported in the literature, such as stone pine (299.2°C), eucalyptus (295.5°C) [23], and the leaf stem of date palm (300°C) [24], suggesting that moxa floss is more combustible and can be ignited more easily than other herbaceous plants. This may explain why moxa floss has remained as the preferred material used for moxibustion over the years.

Moxa floss, as a biomass, has main components of cellulose, hemicellulose, and lignin. In previous studies of biomass pyrolysis, the decomposition of hemicellulose and cellulose mainly occurred at 219.9°C to 314.9°C and 314.9°C to 399.9°C [21, 25–27], while lignin decomposed at a higher and broader temperature range from 160°C to 700°C [27]. The distinct valley observed in the DTG curve at 318.8°C and 323.5°C may be attributed to the thermal decomposition of cellulose, hemicellulose, and to some extent the lignin component.

The last stage of the decomposition process corresponded to the char combustion period in the DTG tail, where the TG curves flattened out at around 500°C. This stage indicates a slower mass loss up to the final temperature and is reflected by the burnout temperature, which is the temperature at which the combustion of samples ceased. The thermochemical decomposition route of a biomass is given as follows: extractives, cellulose, hemicelluloses, and finally lignin or char. The mass loss at this stage is most likely attributed to lignin, fixed carbon, residual volatiles, and char [28].

Based on the DTG curves, the samples of 3 storage years had a higher rate of weight loss as compared to the samples of 10 storage years at the first stage. This implies that the 3 storage year samples have a higher moisture and volatile content as compared to the longer storage year samples. On the other hand, the samples of 10 storage years decomposed at a faster rate in the second stage, in particular sample 5, suggesting lower overall thermal stability. The yield of solid residue was all above 20%, except for sample 5 which was close to zero. These results suggested that sample 5 decomposed more easily and had higher combustibility as compared to other samples.

### 3.2. Py-GC/MS Results

**Figure 3:**
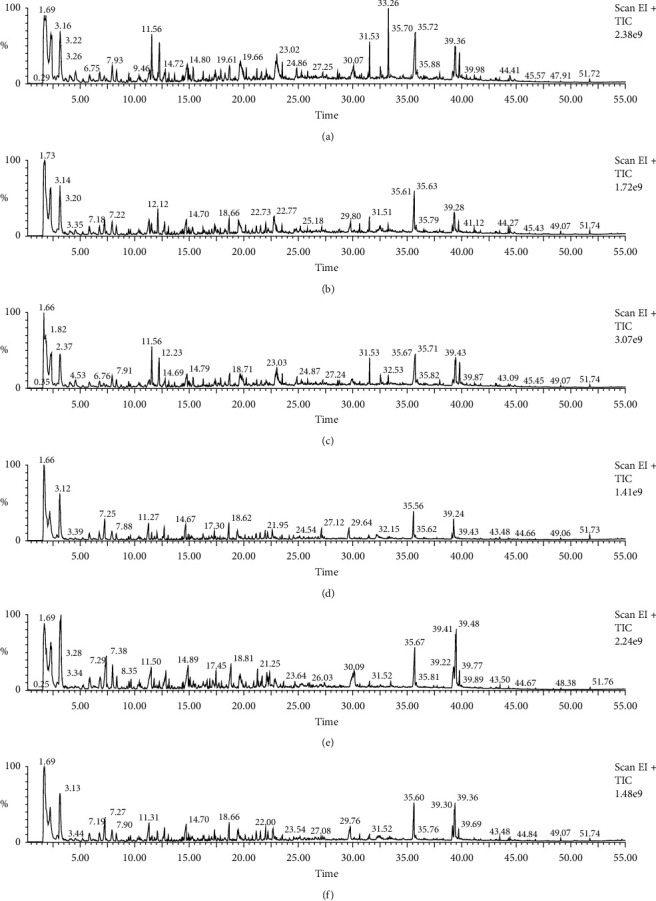
Pyrolysis-GC/MS profiles of the moxa floss samples: (a) sample 1, (b) sample 2, (c) sample 3, (d) sample 4, (e) sample 5, and (f) sample 6.

The pyrolysis profiles of the moxa floss samples obtained at ramped temperature program under 1100°C are shown in Figure 3 and the major peaks of noxious products are identified in Table 3.

Studies on the components of moxa smoke have identified similar compounds such as 1,3-butadiene, benzene, toluene, and naphthalene that are injurious to human health [15, 16]. However, it should be noted that these compounds detected in the pyrolysis products of moxa floss were all relatively low in relative abundance of below 5%. Phenol was detected in all the samples in concentrations between 0.343% and 2.363%. Methyl acetoacetate, an intermediate for common insecticide and bactericide of low toxicity, was detected between 0.377% and 10.03% in all the samples. Interestingly, no volatiles were detected in samples of 10 storage years. This phenomenon may be due to the loss of volatiles during storage in the samples of longer storage years.

## 4. Conclusions

The thermal behavior of moxa floss samples was investigated by thermogravimetric analysis and pyrolysis-gas chromatography/mass spectrometry. The pyrolysis process of moxa floss took place over three stages: (a) the loss of water and volatiles, (b) the decomposition of cellulose and hemicellulose in the second stage, and (c) the decomposition of lignin and char formation in the final stage. Py-GC/MS analysis found noxious products during the combustion and pyrolysis process of moxa floss, but all were of relatively low intensities of below 5% in relative abundance. The low values of ignition temperature suggested that moxa floss is more combustible and can be ignited more easily than other herbaceous plants. This may explain why moxa floss has remained as the preferred material used for moxibustion over the years.

## Figures and Tables

**Table 1:** General description of the selected moxa floss samples in the experiment.

Sample ID	Production area	Storage years	Ratio
1	Ji'nan, Shandong	3	Low
2	Nanyang, Henan	3	Medium
3	Nanyang, Henan	3	High
4	Beijing	10	Low
5	Nanyang, Henan	10	Medium
6	Nanyang, Henan	10	High

**Table 2:** Combustion characteristic parameters of the moxa floss samples.

Sample no.	Mass loss at <120°C (%)	Ignition temperature (°C)	Peak temperature (°C)	Burnout temperature (°C)	Residual weight at 900°C (%)
1	8	217.4	320.6	488.2	23.5
2	8.5	217.2	320.6	486.8	24.2
3	9.9	220.3	315.1	485.4	26.1
4	7.1	228.3	321.6	489.9	21.6
5	4.8	222.2	326.4	492	11.1
6	5.34	217.3	322.6	485.6	22.44

**Table 3:** Identified peaks from Py-GC/MS of moxa floss samples.

Compound	Sample 1		Sample 2		Sample 3		Sample 4		Sample 5		Sample 6	
	RT (min)	RA (%)	RT (min)	RA (%)	RT (min)	RA (%)	RT (min)	RA (%)	RT (min)	RA (%)	RT (min)	RA (%)
Benzene	2.655	0.362			2.623	0.329						
Toluene	4.047	0.834	4.037	0.747								
Benzoic acid	23.021	4.131			14.940	0.383			22.811	0.351		
					23.031	3.876						
Phenol	12.249	2.291	14.950	0.410	12.229	2.363	12.029	0.622	12.249	0.343	12.069	0.77
					15.340	0.870						
Catechol					19.641	3.442			19.661	1.913	19.430	1.760
Hydroquinone			22.791	2.672			22.601	1.084	22.811	0.351	19.690	1.325
Diclofop-methyl							4.137	0.520				
Acetone							9.598	0.339				
Methyl acetoacetate	3.157	5.477	3.137	6.877	3.127	5.208	3.117	7.613	3.227	10.030	6.768	0.970
	6.788	0.647	6.738	0.738	6.758	0.654						
					9.448	0.377						
Acetic anhydride	5.868	0.755	5.838	1.073			5.838	1.151	5.848	1.133		
									6.828	1.315		
Ethyl cyanoacetate	14.930	0.373										
Pimelic acid			17.050	0.395								
5-Aminolevulinic			6.208	0.370								
2-Methylbutane							18.620	1.876				
Furan					2.911	0.451			2.911	0.699	11.589	0.45
					8.318	0.733			11.779	0.422		
Butadiene			8.298	0.722								

Note. RT stands for retention time. RA stands for relative abundance.

## Data Availability

The datasets used in this study are available from the corresponding author upon reasonable request.

## Abbreviations

TGA: Thermogravimetric analysis
DTG: Derivative thermogravimetry
Py-GC/MS: Pyrolysis-gas chromatography and mass spectroscopy
MAHs: Monocyclic aromatic hydrocarbons
PAHs: Polycyclic aromatic hydrocarbons.
